# Factors Driving the Increase in Medical Information on the Web - One American Perspective

**DOI:** 10.2196/jmir.1.1.e3

**Published:** 1999-08-11

**Authors:** Pamela C. Sieving

## Abstract

From the perspective of an academic medical community in the United States, factors driving the increase in medical information on the Internet are examined. These factors are considered in two categories: those that create a demand for information, and those which respond to that demand or attempt to increase or profit by it. The factors explored include demographic, economic, and technological conditions on both sides of the information marketplace. The paper also addresses the responsibilities shared by providers of this information, and possible strategies to assure high-quality resources and informed use of them, both by health care professionals and by patients. The value of informed use is perhaps best conveyed with the following quote.

## Introduction

The most important function of physicians is to help their patients make decisions among competing options of therapeutic interventions."

R.F. Brubaker [1]

As a librarian providing access to information for both patients and the physicians and other health care professionals who work with them, I share with many in the health care and health information fields a deep concern regarding the quality of information on the Internet. More to the point, I am concerned with the quality of information identified and used by patients, their physicians and other health care providers. Information on the Internet exists primarily, without regard to the traditional boundaries of time and place, for which there is limited access, and also of language and information-seeking behaviors. A search on the Web for a medical condition retrieves information from around the world. So while the marketing director of my institution is primarily interested in information that will attract and retain patients in our own clinics, in a sense we also serve patients and the medical staff of our sister institutions around the world.

Ten years ago gophers and CD-ROMs dramatically changed the way we thought about access to information. Today the World Wide Web looks and acts nothing like any medium we have known before. The coincidence of several social, economic and technological changes has led to a unique point in information history, in which there co-exist a greatly increased demand for information and a greatly increased interest on the part of many diverse institutions to fulfill what they perceive to be that need. The resulting increase in medical information, and specifically that available readily and electronically on the Web, is driven by two groups of factors, which can be termed "pull," for those which create a demand for information, and "push," in which providers of information are actively seeking to find users.

## Pull Factors

Pull factors reflect changes in American society which have created a growing, seemingly limitless demand for more access to medical information. The first factor is demographic: an increase in the population of the country, and a shift in age distribution of that population. Overall the population in the United States is predicted to continue to increase, from 151 million in 1950 to 249 million in 1990 with a projected 394 million in 2050 [2,3]. This is due both to birth rates, which continue to more than replace losses by death in our population, and by immigration, particularly from countries in which birth rates are traditionally high-a trend which continues even after arrival in the U.S. Because of political changes, equivalent statistics are not available for all of Europe, but in Germany figures show a different situation: 68.4 million in 1950, 80 million in 1990, with a projected decline to 57.4 million in 2050 [4]. However, in both countries the percentage of those past working age is increasing at a rate higher than that of the overall population, and lifespans are continuing to increase as well. Those 65 and over comprised 8.1% of the U.S. population in 1950, 12.5% in 1990, and are projected to be 18% (70 million) in 2050 [5]. For Germany, the comparable percentages are 14.9%, 21% at present and 37% by 2050 [6,7,8]. Edward Schneider, Dean of the School of Gerontology at the University of Southern California, comments: "The issue that will most affect the quality of life for tomorrow's older population is their future health requirement." [9] The increase in number and proportion of this older population, which is the heaviest user of the medical system, creates two subsidiary pull factors. The first is an increased need for information on which to base medical decisions, and the second is increased discretionary time on the part of this population-although not on the part of health care providers-in which to look for this information.

A second pull factor is a continuing increase in general educational levels attained and literacy rates in the American population. More students graduate from secondary school, the basic level of education funded by all state governments, each year [10]. Consumer book sales are rising at 5% each year [11], with the official literacy rate being close to 98% [12]. One might argue with all of these figures that the general level represented by each of these measures is not as high as could be desired, but compared to any time in our past history, the educational level can be seen only as higher, and on the rise. This creates an increase in the general ability of the population to read and act on information, an increased confidence in doing so, and increased sophistication in evaluating the information and using it to make decisions about medical care.

Increased comfort in dealing with new technologies, particularly those that are computer-based, is a third factor. Half of all American homes now have access to the Internet [13], and the elderly are increasingly willing to try high-tech versions of traditional behaviors [14]. In a recent survey, 67% declared their willingness to "try something new" in computer use [15]. Web use is tripling annually [16]. Nearly all public libraries in the United States now provide both access to and instruction in the use of the Web; it is no longer true that one must invest significant time and financial resources in acquiring and mastering hardware and software. In addition to access in home and libraries, workplace access is growing. One in three American workplace computers had Internet access in 1998 [17]; 30 percent of Americans who categorize at least half of their Internet use as "personal" report that this activity takes place at work [16].

The last thirty years have witnessed a dramatic change in consumerism in the U.S. The publication in 1973 of a slim book, Our Bodies, Our Selves, by a group of feminists in Boston, the Boston Women's Health Book Collective, was one of the first signs that consumers were no longer willing to let physicians dictate medical care. This book, which has just appeared in a new edition for 1999, advocates that women make informed decisions about their own health care, and over the past 25 years has made a great contribution to the information behind those decisions. Also in the early 70's, Americans learned that their government had been involved in an appalling experiment during the 1940s: a group of Black men had been infected with syphilis without their knowledge, in order to study the effects of the disease in a controlled setting. This led to a sense of distrust, particularly by our large minority population groups, of the government as a source of reliable medical advice and help in decision-making, and an element of skepticism being introduced to the public's respect for the medical professions. The film Lorenzo's Oil in 1993 chronicled one family's quest for a treatment for their son's life-threatening illness, and is viewed by some as a model of consumer activism focused on a specific condition. The hospice movement, patterned on a model of end-of-life care that originated in England, is another indication that patients and their families demand to be involved in both medical decision making and actual care. Yet another indication is the increased interest in non-traditional medical care; in the last few years the National Institutes of Health established the Office of Alternative Medicine (now the National Center for Complementary and Alternative Medicine) to fund and promote research in alternative and complementary medicine. Some within the medical establishment view this as a waste of precious resources or worse, but many such therapies are welcomed by patients. The number of Medline searches performed by directly accessing the database at the National Library of Medicine increased from 7 million in 1996 to 120 million in 1997, when free public access was opened; the new searches are attributed primarily to non-physicians [18]. Finally, in late March of 1999 a new site, www.health-mart.net, was announced; this site, under a grant from two agencies of the U.S. government, will attempt to determine the feasibility of providing pricing and outcomes information on 400 diagnoses for essentially all U.S. hospitals to consumers. All of these are indicators of another pull factor: an increase in health care consumerism.

Several dramatic changes within the health care system, perhaps amounting to a revolution, also create a demand for information. In the 1960s, at a time when many were without medical insurance, the national government established two programs to assure access to medical care: Medicare, which provides coverage for services by physicians and hospital stays, and Medicaid, which insures the poor for these same services. Shortly afterwards, health maintenance organizations, which had started in California in the 1940s in very limited numbers, began to seem a viable alternative to fee-for-service care coupled with traditional medical insurance; in HMOs, groups of physicians provide all medical care for a set monthly fee. These organizations were attractive to both employers, who pay most of our medical insurance costs, and patients, who under traditional insurance policies had been responsible for whatever insurance did not cover, because the costs of care were now fixed annually, and known in advance. Now, however, there are concerns that too many of health care providers are primarily concerned with controlling costs rather than caring for patients-that the incentive is to NOT provide medical care-and that patients are not free to choose the best medical care they can identify.

Led by Medicare, under which the government establishes reimbursement rates for every procedure and reimburses physicians and hospitals by diagnosis rather than according to the care an individual patient needs, and by HMOs, which reward physicians for controlling costs, our medical system has dramatically changed. This has affected the care received by those requiring hospitalization. To compare countries in 1998 in Germany the average length of hospitalization for all causes was 11 days [19], whereas the University of Michigan hospitals, which as a tertiary care facility, care for the sickest patients, have reduced the average length of stay to fewer than 6 days in 1998 [unpublished data provided by the Budget Office, University of Michigan Health Care System]. Cost-cutting measures have provoked strong reactions among patients and legislators. The federal government recently passed legislation requiring that women who give birth must be allowed to remain in the hospital for at least 24 hours after delivery. In addition, consumers have successfully fought changes that would have made mastectomy an outpatient procedure. In the United States cataract extraction has been performed only as an outpatient procedure for nearly 15 years, except in rare cases in which the patient is so ill as to require hospitalization for another reason. By contrast, many German hospitals still keep patients in the hospital 3-5 days.

The positive aspect of this change has been a shift in emphasis from treating illness to maintaining and improving health; patients are increasingly viewed as partners with primary responsibility for maintaining their own health. Patient education programs, particularly those emphasizing prevention of illness, consume a new and increasing percentage of the expenditures of all medical care providers. Negatives contributing to the same "pull" on medical information include the fact that fewer nurses are caring for more patients who are sicker for briefer periods of time in the hospital. Figures as high as one nurse for 20 patients are not uncommon [20]. The traditional educational function of nurses, explaining details of care and the medical reasons for that care, is rare today. The cumulative effect of all of these changes is an increased need for information on maintaining and improving health, and on recovering from illness or coping with chronic or terminal disease. Health care providers as individuals are less likely to be the primary source of this information.

Changes in technology are also part of the "pull", enabling consumers of medical information to demand and use the information. Compared to 20 years ago, when personal computers were in their infancy, today's machines are fast and easy to use, requiring much less technical skill on the part of the user. They are cheaper; recently there have been advertisements for "e-tower" machines for less than $500. Even cheaper: "Free PC", a recent development in the States; this is an unsubtle marketing effort, in which free computer, software, and Internet access are provided in exchange for a heavy advertising content every time one accesses the Web. One can also use the Web without actually needing a computer at all: WebTV, now available for less than $100, uses simple equipment and the user's own television monitor and pre-existing telephone lines to access email and the Web. New adaptive technologies are announced almost daily; these enable those with limited visual and manipulative abilities to access the Web with increasing ease.

All of the above create a demand, or pull, for information.

## Push Factors

By contrast, the "push" factors are those initiated by information providers, specifically those on the Web, to meet this demand or create demand where none existed before. Who are they, and what do they want?

1. Governmental Agencies (which have historically had a public-information mission.) The National Library of Medicine and Medline, as well as many other databases, both health and science or technology-related, are prime examples familiar to Americans. Increasingly, websites from the federal government are being used to carry out the government's traditional role of making information freely available. This is simply an extension of a long-established practice in which the public funds its own access to information; the Web makes it quicker and easier in many ways, although the corresponding decrease in printed documents available in what are known as "depository libraries," and available at no charge or nearly so, is of concern to many. The government also provides most funding for clinical and biomedical research in the United States, and is thus also interested in attracting subjects for clinical trials, as well as making the results of research available to patients, medical personnel and other researchers.
2. Medical Health Care Providers who are trying to attract and retain consumers by providing information. This category may include university medical centers, large independent hospitals, and even solo or group medical practices. Additional goals for the investment of time and money in creating and maintaining sophisticated websites include enhancing the image and reputation of the institution, providing information for referring physicians, recruiting research subjects and patients, and attracting students and research associates for the biomedical teaching and research activities of the institution. In the increasingly competitive world of managed care, where the continuing existence of a hospital may depend on its ability to meet the needs of a single large employer such as Ford or Kellogg, an effective Web presence is not a trivial matter.
3. Marketers of Medical Care or Products. This category includes a range of providers, but they are distinguished from the above category by their approach, which involves advertising a highly competitive or perhaps not-yet-accepted therapy, or aggressive marketing of a product. One example of this within the eye care field is LASIK, a laser procedure that reduces or eliminates the need for spectacles or contact lenses. It was only recently approved in the U.S., and is not covered by most medical insurance, so is not subject to price controls. When this procedure was approved in Canada, before the United States, Canadian laser centers marketed this new procedure to the U.S. audience. Another example is ozone therapy for the blinding eye diseases known as retinitis pigmentosa. This very controversial procedure, which costs $15,000 and must be repeated at intervals, is offered primarily in Cuba; the scientific basis for the procedure is unknown. The international nature of the Web allows marketing of these procedures to patients who may be desperate, or merely wealthy. This category may also include commercial operations; perhaps those marketing night vision goggles or other low vision aids, using direct sales approaches aimed primarily at patients as consumers. Commercial information products can also be included here.
4. Libraries. Sometimes discounted simply because they are such a fixture in the U.S. and Europe, libraries, continue to "push" information, in print and audio-visual formats, and increasingly through the Web. Librarians' traditional roles in acquiring, preserving, organizing and disseminating information are changing; their roles in teaching information retrieval and evaluation skills are being enhanced. Librarians may call themselves "cybrarians", but do not fear losing their jobs.
5. Organizations and Support Groups devoted to education and research on diseases and conditions. These vary in their sophistication and legitimacy, but have an obvious presence and appeal on the Web as consumers of health care look for sources of information they can understand and use, and others with whom they can share similar experiences. Individual resources may have the same apparent status as sources when a list of sites is retrieved by one of the search engines.

## Quality vs. Quantity

There are many analogies to dramatize the difficulties faced by health information consumers in retrieving reliable, understandable information in reasonable quantities. One is that using the Internet to look for information is similar to using a firehose to take a drink of water: the virtual flood of information, unsorted, unedited, is of unknown validity and utility. Another is a quote from Molly Mettler, senior vice president of Healthwise, Inc., a non-profit consumer organization, that searching on the Web is

"...like hunting for wild mushrooms. If you know what you're doing and you've got a trusted guide, you can find a real treasure. But you run the chance of picking something toxic." [21].

Not only is the Web unwieldy, but consumers may not be in the best position to understand and evaluate the information they find. A study published in JAMA in February of 1999 described the results of using the Short Test of Functional Health Literacy in an adult population in three U.S. cities. Both Spanish and English speakers were tested in their native language. Approximately 34% of the English speakers, and 54% of the Spanish speakers, were rated "inadequate" or "marginal" users of health information; only 1.6% could correctly interpret instructions on preparing for an upper GI system procedure, while 11.5% were able to correctly interpret instructions on taking medications [22].

We assume that this "medical literacy" is not a problem with professional health care providers, but they face their own challenges. A 1986 study in the Annals of Internal Medicine claimed that if one read two articles per day in the biomedical literature, in one year the reader would be 55 centuries behind on that one year's production [23]. A recent Medline search for asthma-related articles retrieved over 47,000 citations, while an Infoseek search on the Web found over 154,000 sites. Sophisticated medical professionals are often unsophisticated consumers of medical information. The number of competing sources of information makes identifying, evaluating and using new sources of information an increasingly difficult task. One study showed a decline in the knowledge level of general practitioners between five and ten years after the completion of training [24]; this may be a reflection of the gradual loss of previously-learned knowledge, and advances in medical knowledge, coupled with lack of skills for continued learning.

## Strategies

What strategies can be suggested to deal with the real needs of consumers, the "pull" factors described above; the "push" of the needs of producers of information on the Internet; and the associated problems of retrieving, understanding, evaluating and using that information? Several possibilities are suggested in other papers on this issue. Possibilities include:

1. Professional attention to developing meta-sites, such as HealthWeb [http://www.healthweb.org] and their active promotion and marketing, to both consumers and professionals. The open nature of the Web makes it unlikely that any acceptable form of "Web police" will protect anyone from false, misleading or slanted information on the Web.
2. Encouraging development of and reliance on evidence-based medicine. This encourages active evaluation of the existing medical literature, and more critical attention to new clinical research.
3. Teaching of information seeking and knowledge management strategies throughout our educational systems, but particularly for those entering the medical professions. Many academic libraries take this responsibility seriously; the skills may be taught humorously [http://www.improb.com/archives/cat.html] or seriously [http://www.virtualchase.com, for legal resources; http://www.library.ucla.edu/libraries/college/instruct/web/critical.htm for a general approach within the academic community]. A recent study of incoming college students showed that students who cited more sources in their papers, and believed material on the Internet should be viewed with skepticism, had higher grade point averages when compared to their peers [25].
4. Insisting on a clearer understanding of the difference between information technologies and knowledge management strategies.
5. Encouraging and rewarding adherence to Web codes of conduct, such as HON. Easy-to-navigate and reliable sites will be of increasing importance. HON reinforces the importance of principles that are easy to articulate but may nevertheless be overlooked in the preparation of sites.
6. Making a commitment to providing accurate, high-quality links from our own sites seems obvious but is not always acted upon. Individual personal referrals of consumers and patients to good sites are equally important, and are a close corollary to good links.

Theodore Roszak, philosopher and social critic, in a recent New York Times opinion piece, described just why Shakespeare had no need of a word processor, and proceeded to criticize the Web:

The Web is the product of a predatory entrepreneurial sensibility. Like a spider's trap, it exists to ensnare attention with high-tech effects and eye-popping tricks. Those who weave the Web are seeking desperately to transform the medium into the new television... their objective is to lure millions to their sites so they can make lots of money [26].

We hold our own destiny. We can let his words be a warning of the possible judgment of the future on what we do today. If we don't change our direction, we might end up where we're headed.
